# Ca^2+^-dependent nitric oxide release in the injured endothelium of excised rat aorta: a promising mechanism applying in vascular prosthetic devices in aging patients

**DOI:** 10.1186/1471-2482-13-S2-S40

**Published:** 2013-10-08

**Authors:** Roberto Berra-Romani, José Everardo Avelino-Cruz, Abdul Raqeeb, Alessandro Della Corte, Mariapia Cinelli, Stefania Montagnani, Germano Guerra, Francesco Moccia, Franco Tanzi

**Affiliations:** 1 School of Medicine, Department of Biomedicine, Benemérita Universidad Autónoma de Puebla, 13 Sur 2702, Colonia Volcanes, 72000 Puebla, Mexico; 2 Department of Biology and Biotechnology "Lazzaro Spallanzani", Laboratory of Physiology, University of Pavia, via Forlanini 6, 27100 Pavia, Italy; 3 Department of Pharmacology and Therapeutics, Faculty of Medicine, University of Calgary, Calgary, Alberta, Canada; 4 Department of Cardiothoracic Sciences, Second University of Naples, Naples, Italy; 5 Department of Public Health, University of Naples "Federico II", via Pansini 5, 80131, Naples, Italy; 6 Department of Medicine and Health Sciences, University of Molise, via F. De Sanctis, 86100, Campobasso, Italy

## Abstract

**Background:**

Nitric oxide is key to endothelial regeneration, but it is still unknown whether endothelial cell (EC) loss results in an increase in NO levels at the wound edge. We have already shown that endothelial damage induces a long-lasting Ca^2+ ^entry into surviving cells though connexin hemichannels (CxHcs) uncoupled from their counterparts on ruptured cells. The physiological outcome of injury-induced Ca^2+ ^inflow is, however, unknown.

**Methods:**

In this study, we sought to determine whether and how endothelial scraping induces NO production (NOP) in the endothelium of excised rat aorta by exploiting the NO-sensitive fluorochrome, DAF-FM diacetate and the Ca^2+^-sensitive fluorescent dye, Fura-2/AM.

**Results:**

We demonstrated that injury-induced NOP at the lesion site is prevented in presence of the endothelial NO synthase inhibitor, L-NAME, and in absence of extracellular Ca^2+^. Unlike ATP-dependent NO liberation, the NO response to injury is insensitive to BTP-2, which selectively blocks store-operated Ca^2+ ^inflow. However, injury-induced NOP is significantly reduced by classic gap junction blockers, and by connexin mimetic peptides specifically targeting Cx37Hcs, Cx40HCs, and Cx43Hcs. Moreover, disruption of caveolar integrity prevents injury-elicited NO signaling, but not the accompanying Ca^2+ ^response.

**Conclusions:**

The data presented provide the first evidence that endothelial scraping stimulates NO synthesis at the wound edge, which might both exert an immediate anti-thrombotic and anti-inflammatory action and promote the subsequent re-endothelialization.

## Background

Endothelial injury is regarded as the early event that leads to the onset and progression of severe vascular disorders, such as thrombosis, hypertension, and atherosclerosis [1]. Endothelial cell (EC) loss physiologically occurs due to physiological turnover in focal areas of the inner surface of blood vessels which are adjacent to regions with low or absent replication [2,3]. When the extent of EC loss is such small, i.e. limited to a belt of cells around the whole circumference of the arterial vessel, the re-endothelization process is driven by proliferation, spreading, and migration of surviving ECs into the damaged site [4]. On the other hand, larger areas of injury may release chemical messages, such as concentration gradients of vascular endothelial growth factor (VEGF) and stromal derived factor-1α (SDF-1α), that recruit circulating endothelial progenitor cells to the wound edge in order to replace damaged endothelium [5-10]. The break in the anatomic integrity of vascular endothelium is significantly larger in subjects undergoing medical interventions, such as deployment of endovascular devices and percutaneous transluminal coronary angioplasty [2,11,12]. Such a heavy loss of ECs from the vascular wall dampens the beneficial effects of reconstructive surgery and prompts the quest for pharmacological treatments aiming at restoring the continuity of endothelial monolayer [2,11-13]. Accordingly, the de-endothelialisation of arterial vessels may cause thrombi formation and neointimal hyperplasia, giving raise to a process termed as "in-stent restenosis" (ISR) which renarrows the arterial lumen [2,11,12]. Drug-eluting stents (DES) may be implanted during or after angioplasty to inhibit neointimal hyperplasia and prevent ISR [2,12,14]. Unfortunately, the most commonly employed stents deliver drugs, such as sirolimus and paclixatel, that cause a long-term inhibition of endothelial proliferation and migration. These untoward off-target effects significantly delay endothelial regrowth, thereby leaving uncovered the surface of the stent and increasing the risk of late in-stent thrombosis [14,15].

Aging is accompanied by a decline in the healthy function of multiple organ systems, leading to increased incidence of mortality from many diseases. Oxidative stress and elevated ROS (Reactive oxygen species) has been implicated in the mechanism of senescence and aging; they are also involved in cancer, diabetes, neurodegenerative, cardiovascular and other diseases[16,17] Overproduction of oxidant molecules is due to several stress agents such chemicals, drugs, pollutants, high-caloric diets and exercise[18].

Nitric oxide (NO), a gasotransmitter that may be synthesized and released by ECs [1,19], might play a key role in healing injured endothelium. Accordingly, NO inhibits apoptosis and enhances EC proliferation, migration, and tubulogenesis [19]. Moreover, NO might serve as anti-inflammatory signal at the injured site by preventing local platelet activation and thrombus formation, by causing vasorelaxation, and by inhibiting the phenotypic switch of vascular smooth muscle cell (VSMC) [1,19]. Restoration of NO production at the site of vascular injury may attenuate neointimal hyperplasia and adverse the onset of the atherosclerotic process [20]. In vascular endothelium, NO may be synthesized by two different isoforms of NO synthase (NOS), namely inducible NOS (iNOS/NOS2), which mediates NO liberation during inflammatory reactions, and endothelial NOS (eNOS/NOS3), which is preferentially recruited by stimuli [19,21]. The catalytic reaction consists in the conversion of L-arginine to L-citrulline and requires several cofactors, such as NAPDH, tetrahydrobiopterin and O_2 _[19]. Unlike iNOS, which is inducible and Ca^2+^-independent, both iNOS and eNOS are constitutive and bear a calmodulin-binding site whose binding to cytosolic Ca^2+ ^is essential to stimulate NO synthesis [19]. In several types of ECs, eNOS is preferentially recruited by Ca^2+ ^store-operated Ca^2+ ^entry (SOCE), which is gated by depletion of the inositol-1,4,5-trisphosphate (InsP_3_)-sensitive stores within the endoplasmic reticulum (ER) [8,22]. This feature is due to the close proximity between eNOS and SOC channels at the caveolae, cholesterol-enriched surface microdomains that compartmentalize signal transduction molecules [23]. Cholesterol-binding drugs, such as methyl-β-cyclodextrin (MβCD), have been used to disrupt caveolae and impair NO signalling in vascular endothelium [24]. That NO is involved in EC response to injury has been suggested by the elevation in both eNOS protein and enzyme activity in the regenerating endothelium of rat aorta [25]. Moreover, a recent study carried out on this preparation reported an increase in [Ca^2+^]_i _in ECs nearby the lesion site [2,9,26,27]. The Ca^2+ ^response to injury comprises an initial peak, mainly due to Ca^2+ ^release from InsP_3_-sensitive receptors (InsP_3_Rs), which is followed by a long-lasting decay phase mediated by Ca^2+ ^entry across the plasma membrane [27]. InsP_3 _synthesis requires phospholipase C (PLC) activation by ATP (or ATP-derived nucleotides) released from ruptured cells [27]. Ca^2+ ^inflow is supported by connexin (Cx) hemichannels (CxHcs), which have recently been described as alternative Ca^2+ ^entry routes in vascular ECs as well as other non-excitable cell types [26-31]. Cxs may be classified on the basis of the molecular weight with three main isoforms (Cx37, Cx40, and Cx43) being found in ECs from several vascular beds, including rat aorta [32,33]. Despite the available evidences show an increase both in eNOS activity and in [Ca^2+^]_i _within lesioned endothelium, there is no report of injury-induce NO release in blood vessels. Understanding the signal transduction machinery leading to endothelial activation and NO synthesis might be clinically relevant to design alternative strategies to restore endothelial integrity and prevent ISR.

In the present investigation, we sought to determine whether NO synthesis occurs in ECs facing the injury site of excised rat aorta. We further aimed at elucidating the signal transduction pathway responsible for NO release by lesioned endothelium. These goals were accomplished by loading ECs with Fura-2/AM, a Ca^2+^-sensitive fluorochrome, and DAF-FM diacetate, a NO-sensitive fluorescent dye. We provided, for the first time, the evidence that mechanical injury induces a long lasting NO production (NOP) in cells nearby the lesion site which requires Ca^2+ ^entry through uncoupled Cx37Hcs, Cx40Hcs, and Cx43Hc. These results might aid in developing novel pharmacological treatments devoted to restore endothelial integrity upon invasive medical interventions.

### Methods

#### Dissection of the aorta

Wistar rats aged 2-3 months were sacrificed with an overdose of diethyl ether. The thoracic and abdominal aorta were dissected out and perfused with physiological salt solution (PSS). The vessel was cleaned of the surrounding connective tissue, cut in ~5 mm long rings, stored in PSS at room temperature (22-24 °C) and used within 5 hours. All the animal protocols were approved by the East Tennessee State University's Animal Care and Use Committee. All experiments conform to the Guide for the Care and Use of Laboratory Animals published by the US National Institutes of Health (NIH Publication No. 85-23, revised 1996). The animal handling was under the continuous control of the Veterinary Surgeon of the University of Pavia.

#### Solutions

PSS had the following composition (in mM): 150 NaCl, 6 KCl, 1.5 CaCl_2_, 1 MgCl_2_, 10 Glucose, 10 Hepes. In Ca^2+^-free solution (0 Ca^2+^), Ca^2+ ^was substituted with 2 mM NaCl, and 0.5 mM EGTA was added. Solutions were titrated to pH 7.4 with NaOH. Aortic rings were bathed in 0 Ca^2+ ^for no longer than 90 sec before inducing the injury. Control experiments have demonstrated that such a short pre-incubation period is not able deplete intracellular Ca^2+ ^stores [27].

[Ca^2+^]_i _and NO measurements

The technique used to evaluate changes in [Ca^2+^]_i _in intact endothelium has been previously described [26,27,34-37]. The aortic ring was opened and loaded with 16 µmol Fura-2/AM for 60 min at room temperature, washed and fixed by small pins with the luminal face up. *In situ *ECs were visualized by an upright epifluorescence Axiolab microscope (Carl Zeiss, Oberkochen, Germany), equipped with a Zeiss ×63 Achroplan objective (water-immersion, 2.0 mm working distance, 0.9 numerical aperture). ECs were excited alternately at 340 and 380 nm, and the emitted light was detected at 510 nm. The exciting filters were mounted on a filter wheel (Lambda 10, Sutter Instrument, Novato, CA, USA). Custom software, working in the LINUX environment, was used to drive the camera (Extended-ISIS Camera, Photonic Science, Millham, UK) and the filter wheel, and to measure and plot on-line the fluorescence from 10-15 rectangular "regions of interest" (ROI) enclosing one single cell. [Ca^2+^]_i _was monitored by measuring, for each ROI, the ratio of the mean fluorescence emitted at 510 nm when exciting alternatively at 340 and 380 nm (shortly termed "ratio"). An increase in [Ca^2+^]_i _causes an increase in the ratio. Ratio measurements were performed and plotted on-line every 5 s. The experiments were performed at room temperature (21-23 °C).

NO production in ECs in intact rat aorta was monitored with the membrane permeant 4-Amino-5-methylamino-2',7'-difluorofluorescein (DAF-FM) diacetate. Once open, the aortic ring was fixed by small pins with the luminal side facing up, loaded with 10 µM DAF-FM for 60 min at room temperature and washed in PSS for one hour. DAF-FM fluorescence was measured by using the same equipment described for Ca^2+ ^recordings but with a different filter set, i.e. excitation at 480 nm and emission at 535 nm wavelength (emission intensity was shortly termed "NO_i_"). NO measurements were performed and plotted on-line every 5 s. Again, off-line analysis was performed by using custom-made macros developed by Microsoft Office Excel software. The experiments were performed at room temperature. DAF-FM is essentially non-fluorescent until it irreversibly reacts with the nitrosonium cation produced by spontaneous oxidation of newly synthesized NO. The resulting fluorescent compound is trapped in the cytoplasm, so that DAF-FM fluorescence summates with continual NO production. As also found in bovine aortic ECs [38], DAF-FM fluorescence in rat aortic ECs underwent a persistent and linear increase likely due to the basal NO synthesis [19]. Accordingly, the change in baseline fluorescence was dramatically reduced when aortic rings were pre-incubated with L-NAME (5 mM), which inhibits eNOS by competing with its natural substrate, L-arginine (data not shown), or in 0Ca (data not shown). This observation was somehow expected since aortic endothelium has long been known to regulate vascular tone by basal release of NO [19]. Therefore, for each experiment, the basal DAF-FM signal was recorded for at least 20 minutes and the resulting curve fitted off-line by a linear regression equation in order to calculate the slope (i.e. the rate) of basal fluorescence increase. The slope was subtracted from the recorded trace, thus enabling to measure S-Nitroso-N-acetylpenicillamine (SNAP)-, ATP- and injury-induced NOP independently on basal eNOS activity [39] (see below). NO synthesis was evaluated by subtracting the baseline level (1 min before the increase in DAF-FM fluorescence) to the average value of the plateau phase (1 min before the end of the recording) recorded upon either ATP administration or endothelial scraping.

**Figure 1:**
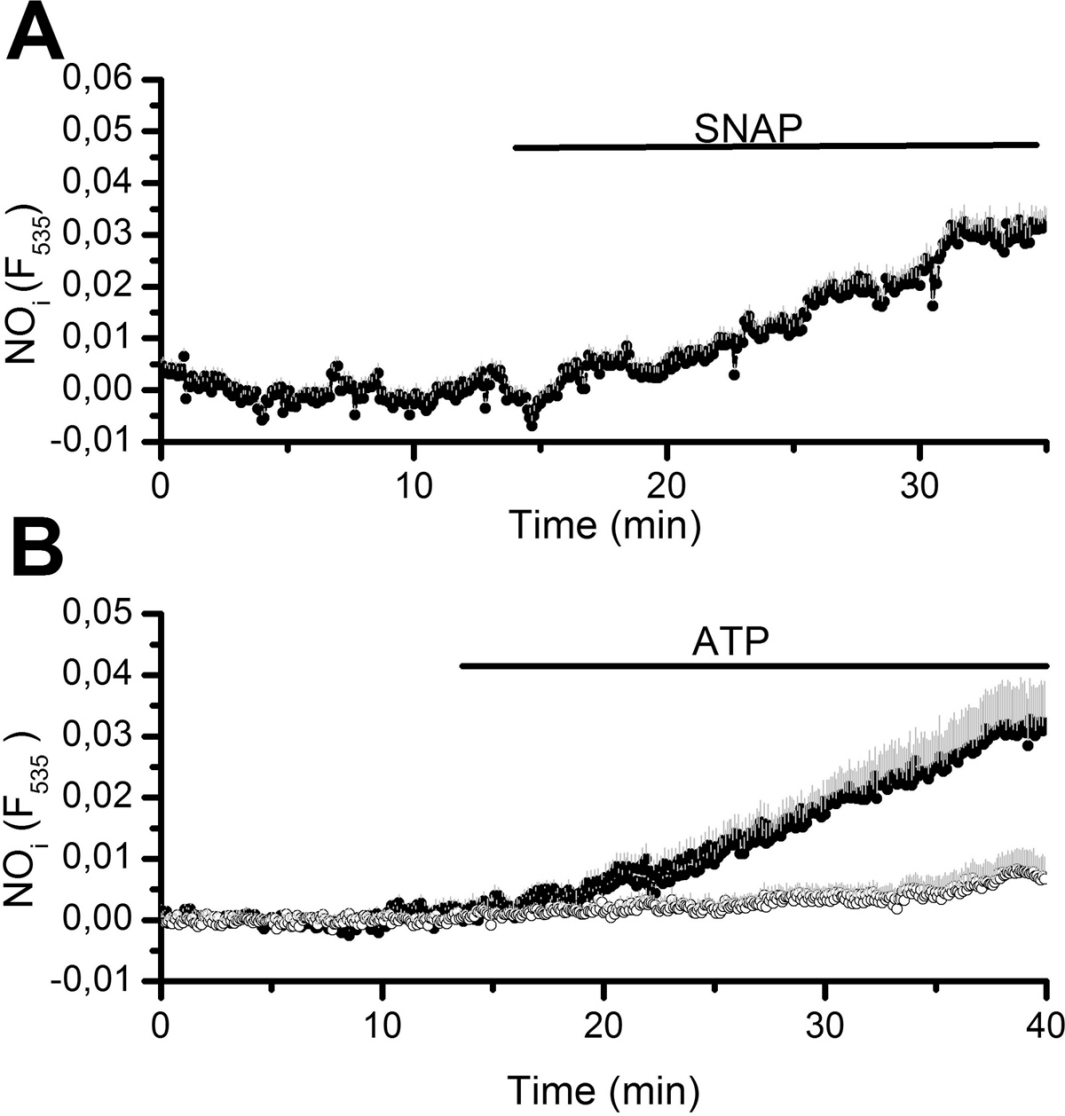
**NO production in rat aortic endothelium**. A, exposition to SNAP (500 μM) caused an increase in DAF-FM fluorescence in native rat aortic endothelium *in situ*. The trace is the mean±SE of 11 cells from the same visual field. B, ATP (300 μM) induced an elevation in intracellular NO (closed circles) that was dramatically hampered when the rings were pre-treated with L-NAME (5 mM) for 40 min (white circles). The traces are the mean±SE, respectively, of 10 (PSS) and 8 (L-NAME) cells from two aortic rings harvested from the same animal on the same day.

In order to assess the ability of the fluorimetric set up to detect evoked changes in intracellular NO levels, aortic rings were exposed to the NO donor, SNAP, and ATP, which has been shown to elicit NO production in a variety of ECs from different vascular districts [38,40]. As shown in Figure 1A and Figure 1B, respectively, SNAP (500 µM) and ATP (300 µM) caused an increase in the intensity of DAF-FM fluorescence in 19 out of 19 cells and in 170 out of 170 cells, respectively. On average, L-NAME (5 mM) reduced by 86% ATP-induced NO production (0.025 ± 0.001, *n *= 170, *vs*. 0.0041 ± 0.0009, *n *= 192, p < 0.001) (Figure 1B).

#### Mechanical disruption of ECs

As shown in [26,27], aortic endothelium was injured under microscopic control by means of a glass microelectrode with a broken tip of about 30 µm diameter, driven by an XYZ hydraulic micromanipulator (Narishige, Japan). Images of either Fura-2 or DAF-FM/Fura-2 loaded ECs, together with numbered ROIs, were taken before the lesion, in order to identify the cells facing the injury site. As aforementioned, the dissection procedure could itself damage the intimal layer and cause SMCs to be loaded with Fura-2. These rings were, therefore, discarded. The microelectrode was first positioned almost parallel and very near to the endothelium surface. It was then moved downward, along the Z-axis, until the electrode tip gently touched the endothelium, and moved horizontally across the visual field to scrape 1-3 consecutive rows of ECs along the whole diameter of the aortic ring. The following lesion was 170 μm long and 20 μm wide. This procedure, which allowed monitoring of NO signals in ECs adjacent to the lesion, mimics the ablation of the endothelial lining achieved in pre-clinical studies addressing the cellular mechanisms of intimal regrowth upon stent deployment [41-43]. The scraping of the endothelial monolayer imposed a physical stretching on the underlying SMCs which resembles the condition occurring during clinical interventions. Ethidium bromide (EB), a fluorescent molecule unable to cross an intact plasma membrane and therefore indicative of damaged/dead cells, was used to check the viability of both ECs nearby the injury and underlying SMCs [27]. When smooth muscle fibers were lesioned by mechanical scratching and stained by EB, the experiments were discarded.

#### Data analysis

For each protocol, data were collected from at least three rats. The amplitude of the peak Ca^2+ ^response was measured as the difference between the ratio at the peak and the mean ratio of 1 min baseline before the peak. The details of NO measurements have been reported above. Only cells residing in the first row nearby the wound were used to elaborate the average value. For Ca^2+ ^and NO measurements, statistical comparisons were made by Student's *t*-test for unpaired observations.

#### Chemicals

Fura-2/AM and DAF-FM was obtained from Molecular Probes (Molecular Probes Europe BV, Leiden, The Netherlands). N-(4-[3,5-bis(trifluoromethyl)-1H-pyrazol-1-yl]phenyl)-4-methyl-1,2,3-thiadiazole-5-carboxamide (BTP-2) was purchased from Calbiochem (La Jolla, CA, USA). All other chemicals were purchased from Sigma. Cx-mimetic peptides, including ^37,43^Gap27 and ^40^Gap27, were synthesized by Severn Biotech (Kidderminster, UK); purity was >95%. In more detail, ^37,43^Gap27 and ^40^Gap27 mimic a highly conserved SRPTEK sequence present in the second extracellular loop of Cxs 37 and 43 and of Cx 40, respectively [15,19]. Their scrambled versions were also synthesized by Severn Biotech. All other chemicals were of analytical grade and obtained from Sigma.

### Results

#### Injury augments intracellular NO levels in the intact endothelium of rat aorta

**Figure 2:**
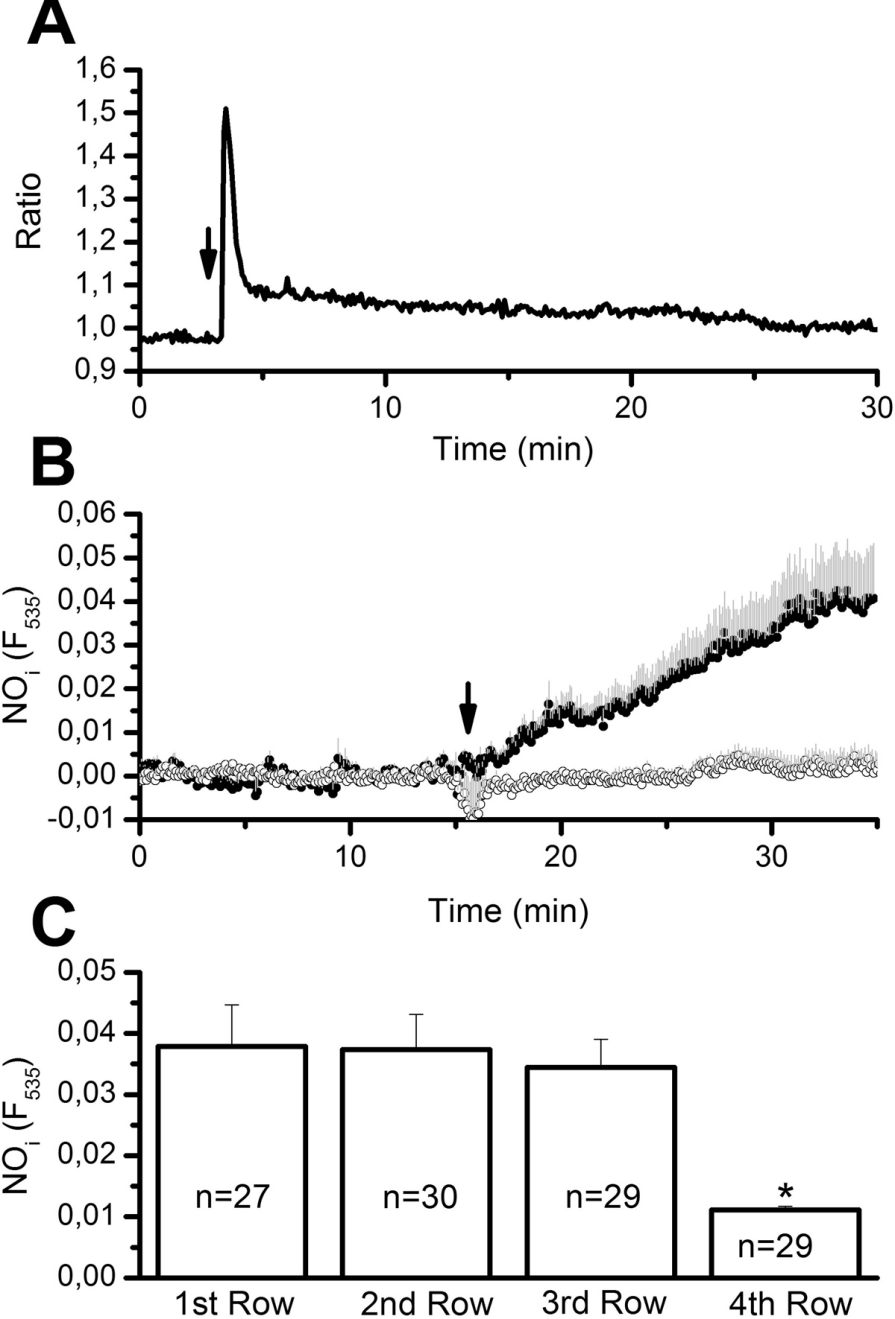
**NO synthesis induced by mechanical injury rat aortic endothelium**. A, Ca^2+ ^signal elicited by lesioning rat aorta in PSS. The trace, recorded from a single cell, is representative of 10 cells from the same visual field. B, injury induced an elevation in intracellular NO levels that was abolished when the cells were pre-treated with the eNOS inhibitor, L-NAME (5 mM), for 40 min. The traces are the mean±SE, respectively, of 10 (PSS, closed circles) and 5 (L-NAME, open circles) cells from two aortic rings harvested from the same animal on the same day. C, mean±SE of the NO signal recorded at increasing distance from the lesion site. In this and the following figures, the arrow indicates when injury is performed. Asterisk indicates a level of significant difference <0.05.

**Figure 3:**
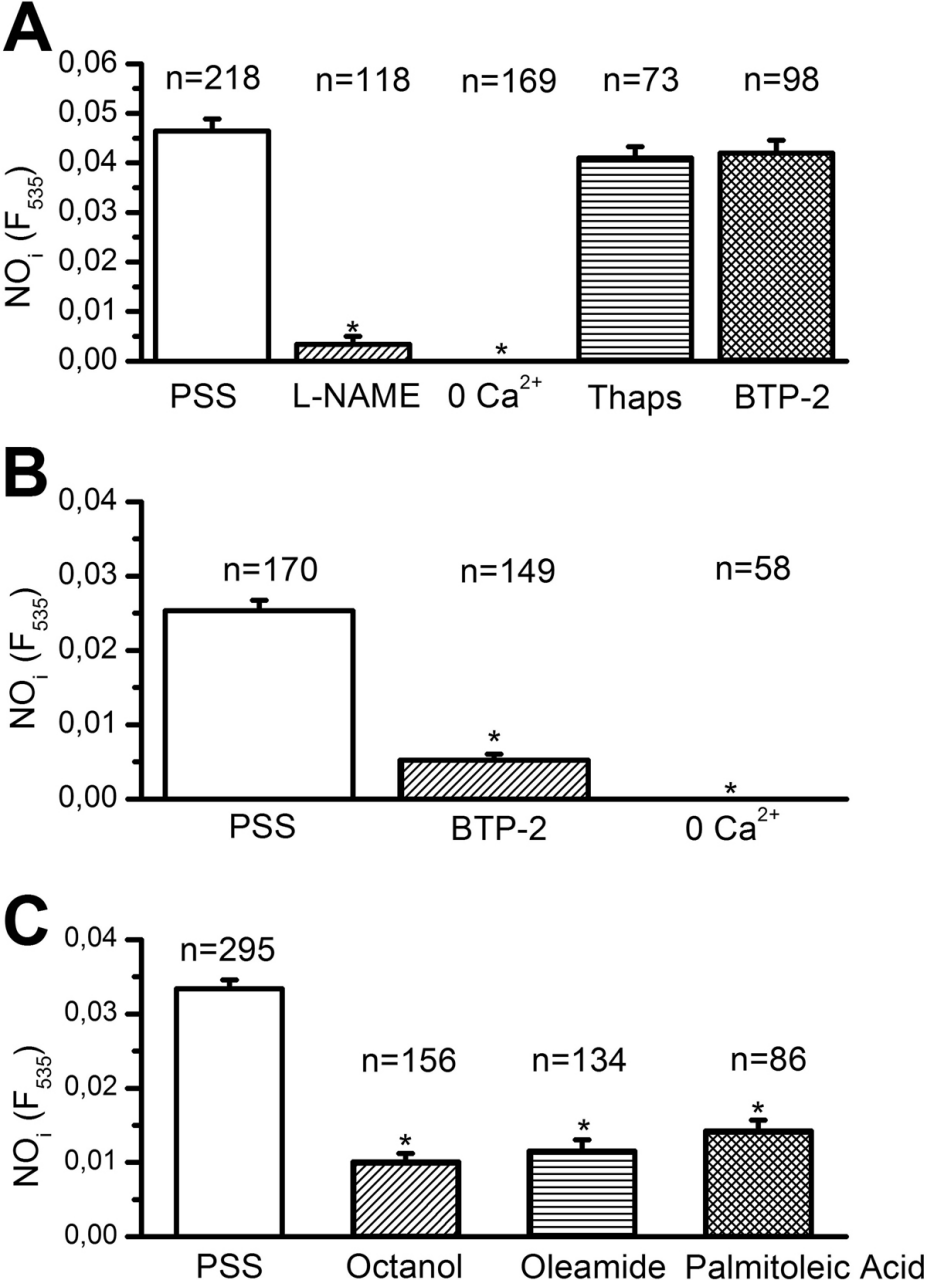
**Amplitude of the NO response to injury (A and C) and ATP (B) following the designated treatments**. The asterisk indicates a level of significant difference <0.05. The number of ECs analyzed in each condition, harvested from 3-9 animals, is indicated above the bars. See the text for drug concentrations.

Figure 2A depicts the injury-induced increase in [Ca^2+^]_i _occurring within the ECs adjacent to the lesion site and which have lost their contact with the scraped cells. The Ca^2+ ^signal consisted in an initial transient peak, mainly due to InsP_3_-dependent Ca^2+ ^release, followed by a prolonged decay phase caused by Ca^2+ ^entry from the extracellular milieu [27]. When the damage was performed on an aortic ring loaded with DAF-FM, it induced a gradual increase in fluorescence that reached a plateau after about 20 min (Figure 2B). This feature is consistent with the concomitant long-lasting Ca^2+ ^inflow [27], which might sustain NO synthesis [19,38]. In the presence of the eNOS inhibitor, L-NAME (5 mM), mechanical damage failed to augment intracellular NO (Figure 2B). The statistical analysis is reported in Figure 3A. Similar to the intercellular Ca^2+ ^wave, which did not spread farther than the 4^th ^raw of cells, the amplitude of the NO signal significantly (p < 0.05) decayed at this location (Figure 2C). This feature hints at a strong correlation between the increase in [Ca^2+^]_i _triggered by the injury and the accompanying NO synthesis.

#### Injury-induced NOP depends on extracellular Ca^2+ ^entry

It has long been known that, in vascular endothelium, eNOS is selectively activated by Ca^2+ ^inflow rather than intracellular Ca^2+ ^release [8,22,40]. When mechanical damage was carried out in absence of extracellular Ca^2+^, a maneuver which abolished the prolonged decay-phase of the Ca^2+ ^signal, the injury did not result in a detectable NOP (Figure 3A). The involvement of intracellular Ca^2+ ^mobilization was investigated with the aid of thapsigargin (2 μM), an inhibitor of the endoplasmic reticulum Ca^2+^-ATPase, which prevents Ca^2+ ^re-uptake into the stores and leads to their depletion. Preliminary experiments showed that pre-incubating the rings for 30 min with 2 μM thapsigargin abolished the intracellular Ca^2+ ^mobilization triggered by supramaximal concentrations of ATP (300 μM) (*n *= 79; data not shown). Therefore, such a treatment causes the depletion of the Ca^2+ ^reservoir underlying the initial Ca^2+ ^response to injury [27]. The NO signal induced by endothelial scraping, however, was not significantly affected by thapsigargin (Figure 3A). Overall, these results strongly suggest that injury-promoted NOP in surviving ECs is sustained by Ca^2+ ^entry, while is insensitive to Ca^2+ ^release from ER. SOCE represents the preferential route for extracellular Ca^2+ ^to engage eNOS in ECs [8,22,40]. However, BTP-2 (20 μM), a widely employed blocker of store-dependent Ca^2+ ^inflow [6,8], does not significantly affect injury-induced NO synthesis (Figure 3A). We subsequently focused on the response to ATP, which elicits a BTP-2-sensitive SOCE in rat aortic endothelium [27]. ATP-induced NO synthesis was strongly inhibited in 0 Ca^2+ ^(Figure 3B) and in the presence BTP-2 (Figure 3B). Collectively, these data concur with our previous findings on injury-induced Ca^2+ ^inflow and rule out a detectable role for SOCE in NO release by lesioned endothelium.

#### Gap junction inhibitors reduce NO synthesis induced by Ca^2+ ^entry in injured endothelium

**Figure 4:**
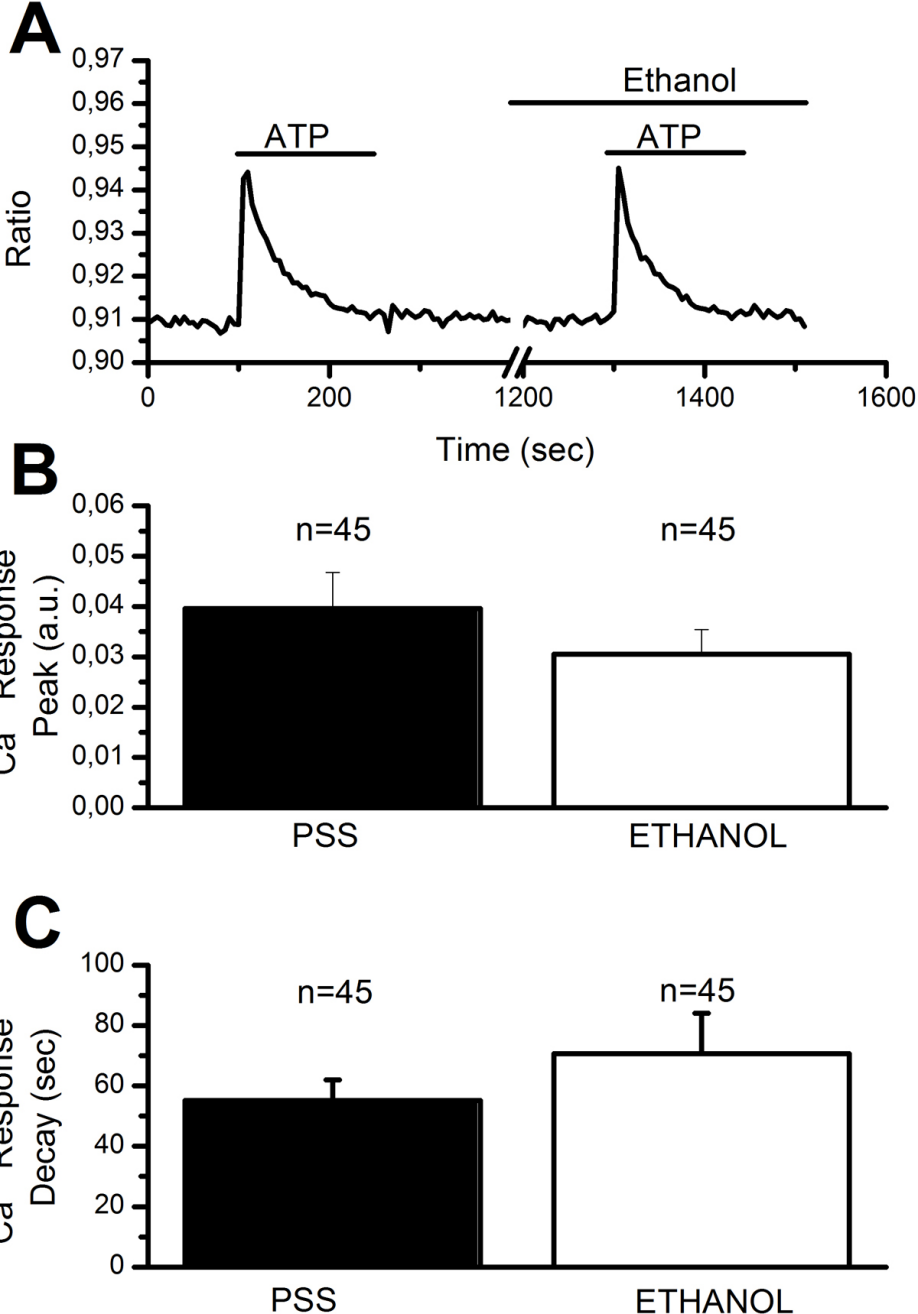
**Ethanol does not significantly affect the Ca^2+ ^response to ATP**. A, 20 min pre-incubation with octanol (4 mM) does not remarkably affect either the amplitude or the kinetics of the Ca^2+ ^response to ATP (20 μM). The trace is the average of 16 cells from the same microscopic field of view. B, mean±SE of the peak of ATP-induced elevation in [Ca^2+^]_i_. The asterisk indicates a level of significant difference <0.05. The number of ECs analyzed in each condition is indicated above the bars. C, B, mean±SE of the rate of decay of ATP-induced Ca^2+ ^signal. The decay was measured by fitting the Ca^2+ ^tracings with a first order exponential equation (OriginPro 7, OriginLab Corp, Northampton MA). The asterisk indicates a level of significant difference <0.05. The number of ECs analyzed in each condition is indicated above the bars.

The long-lasting decay phase of injury-induced Ca^2+ ^elevation in rat aortic rings is dramatically diminished by gap junction blockers, such as octanol, palmitoleic acid and oleamide [26,27,44,45]. In agreement with the Ca^2+ ^measurements [27], octanol (4 mM), oleamide (200 μM), and palmitoleic acid (50 μM) dramatically affected NO synthesis elicited by endothelial scraping (Figure 3C). In the light of our previous observations [27], these findings hint at a role for Ca^2+ ^inflow through CxHcs in mediating NOP in injured endothelium. We assessed the specificity of gap junction blockers by measuring the Ca^2+ ^response to ATP (20 μM) in the presence of octanol. At this dose, ATP evokes an InsP_3_-dependent Ca^2+ ^release, which is subsequently buffered by the combined action of SERCA and Na^+^/Ca^2+ ^exchanger [27,34,46]. As shown in Figure 4, pre-incubating rat aortic rings with 4 mM octanol for 20 min did not significantly affect either the magnitude of the [Ca^2+^]_i _elevation or the rate of its decay phase to the baseline. This result concurs with previous evidence about the efficacy of gap junction blockers as selective uncoupling drugs in rat aortic rings [47,48], and further support the role of CxHcs in mediating NO synthesis in injured endothelium.

#### Effect of Cx-mimetic peptides on injury-induced Ca^2+ ^elevation and NO production

**Figure 5:**
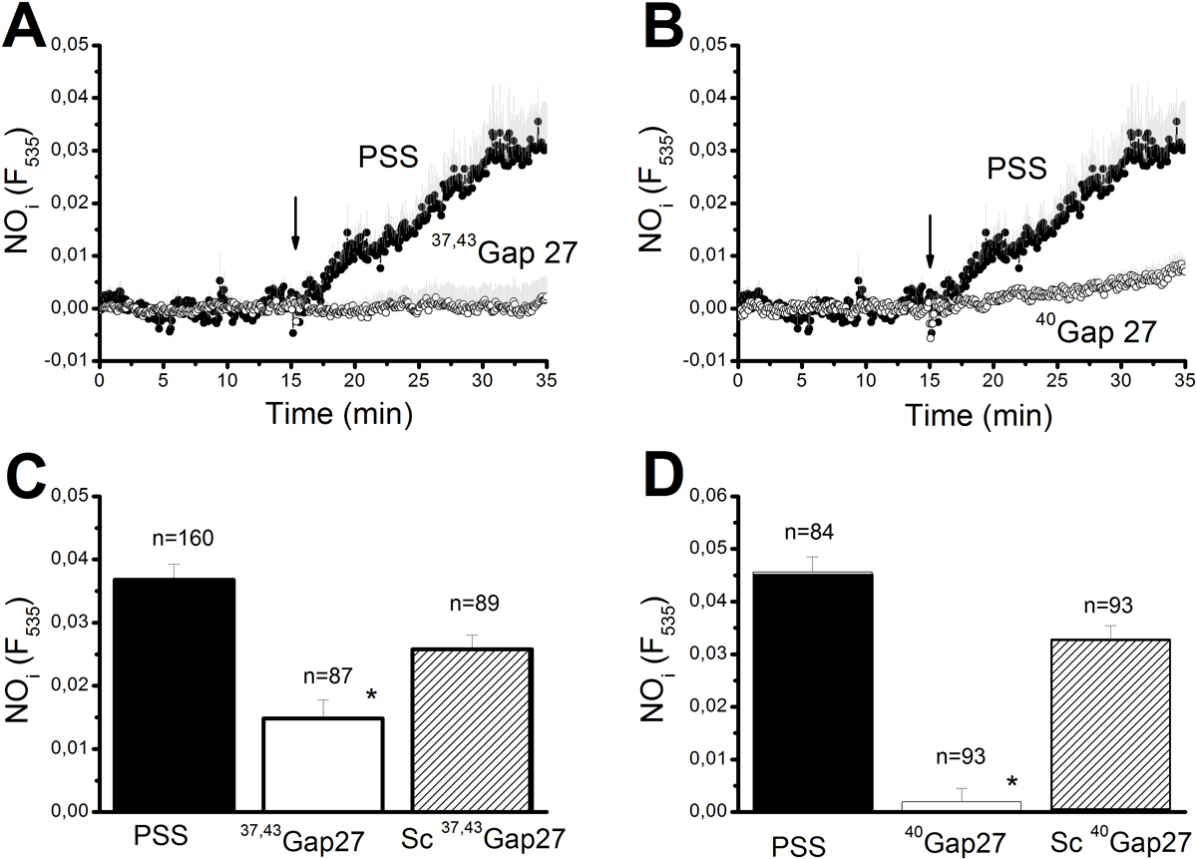
**Connexin-mimetic peptides affect injury-induced NO synthesis**. 1 hour pre-incubation with either ^37,43^Gap27 (300 μM) (A) or ^40^Gap27 (300 μM) (B) dramatically attenuated the increase in intracellular NO levels provoked by endothelial lesion. For sake of clarity, the control curve has been depicted in each Panel. All the experiments depicted in this figure have been carried out on the same day on different rings isolated from the same animal. The control (PSS, closed circles) trace is the mean±SE of 9 cells, while the ^37,43^Gap27 (open circles in Panel A) and ^40^Gap27 traces (open circles in Panel B) are, respectively, the mean±SE of 5 and 7 cells. C and D, mean±SE of NOP elicited by injury following the designated treatments. Each drug was applied was 20 min before carrying out the lesion. The asterisk indicates a level of significant difference <0.05. The number of ECs analyzed in each condition is indicated above the bars. See the text for drug concentrations

**Figure 6:**
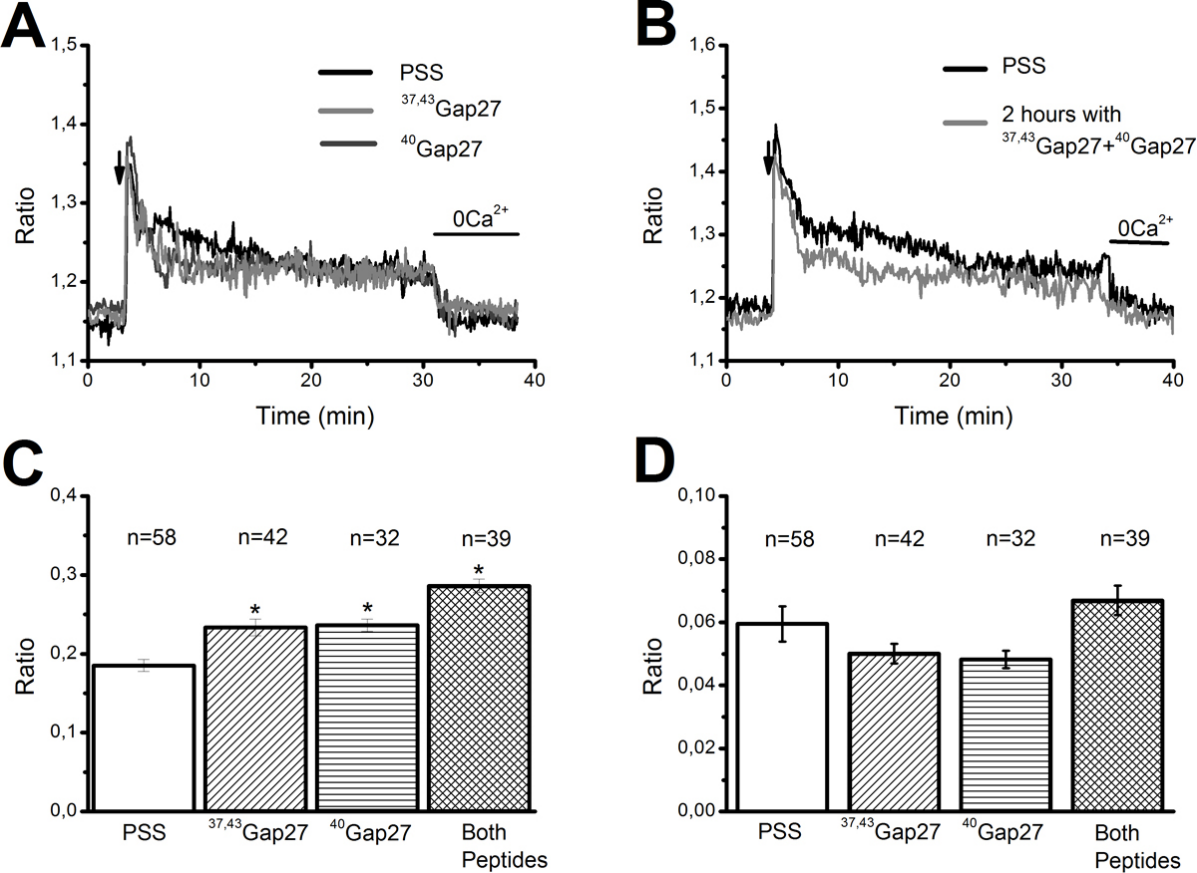
**Connexin mimetic peptides do not impair injury-induced elevation in [Ca^2+^]_i _in rat aortic endothelium**. A, 1 hour pre-incubation with either ^37,43^Gap27 (300 μM) (gray trace) or ^40^Gap27 (300 μM) (dark grey trace) did not reduce injury-elicited intracellular Ca^2+ ^signals in native endothelium of rat aorta. B, 2 hour pre-incubation with both ^37,43^Gap27 (300 μM) and ^40^Gap27 (300 μM) (grey trace) did not impair the intracellular Ca^2+ ^response to injury at wound edge of rat aortic endothelium. C and D, mean±SE of NOP elicited by injury following the designated treatments. The asterisk indicates a level of significant difference <0.05. The number of ECs analyzed in each condition is indicated above the bars.

Rat aortic endothelium may express three Cx isoforms, namely Cx37, Cx40, and Cx43 [32,49], albeit a recent work reported the presence of Cx32 in various cultured human ECs [50]. The contribution of each Cx isotype to injury-evoked NO liberation was examined by exploiting Cx-mimetic peptides containing amino acid sequences corresponding to the Gap27 extracellular domain of Cx37 and Cx43 (^37,43^Gap27) and to the Gap27 extracellular domain of Cx40 (^40^Gap27). Although initially designed to impair gap junctional communication, the primary target of these compounds is provided by CxHcs. The extracellular-loop sequence that interacts with the peptides is freely available in the CxHc form [51], so that full blockade of the unpaired hemichannel requires a much shorter pre-incubation (minutes *vs*. hours or longer) and much lower doses (micromolar *vs*. millimolar range) than inhibition of gap junctional coupling [51]. These compounds have been found to selectively inhibit Cx-dependent signalling in a variety of cell types, including rat aortic ECs [49,52], when the genetic suppression of specific Cx isoforms is not feasible, such as in rat endothelium [33,45]. In addition, Cx-mimetic peptides do not present the drawbacks reported in Cx-deficient mice [53]. For instance, Cx40^-^/^- ^mice manifest either higher [54] or lower [55] levels of Cx37 as well as a significant decrease in Cx43 expression [56]. This feature makes the interpretation of the physiological data collected from these animals difficult. Incubating the aortic rings for 1 hour with either ^37,43^Gap27 (300 μM) or ^40^Gap27 (300 μM) significantly (p < 0.05) reduced injury-evoked NOP (Figure 5A and Figure 5B). The statistical analysis of these experiments has been reported in Figure 5C and Figure 5D. NO signalling was unaffected by peptides corresponding to scrambled sequences in Gap27, indicating that the inhibition resulted from specific sequence recognition on the selected Cx isotypes (Figure 5C and Figure 5D). Therefore, it is reasonable to conclude that Ca^2+ ^entry driving eNOS activity and NO liberation at the wound edge is supported by Cx37Hcs, Cx40Hcs, and Cx43Hcs. As discussed elsewhere [57], eNOS activation requires a sub-plasmalemmal Ca^2+ ^increase rather a bulk increase in cytosolic Ca^2+ ^levels. Such a localized Ca^2+ ^event may not even be detected by conventional epifluorescence microscopy [58]. As a consequence, we went on in assessing whether Cx-mimetic peptides targeting Cx37, Cx40, and Cx43 were also able to reduce the long-lasting decay phase of the Ca^2+ ^response to mechanical damage. Figures 6A to 6D depict that neither ^37,43^Gap27 (300 μM) nor ^40^Gap27 (300 μM) significantly reduce either the initial Ca^2+ ^peak or the subsequent decay phase. Rather, both Cx-mimetic peptides increased the amplitude of the initial Ca^2+ ^response (Figure 6A-6C). Consistently, acute application of both peptides during recovery of the Ca^2+ ^signal towards the baseline did not decreased the [Ca^2+^]_i _(*n *= 31, ^40^Gap27; *n *= 52, ^37,43^Gap27) (not shown). The same results were found by extending the pre-incubation period with both ^37,43^Gap27 and ^40^Gap27 up to 2 hours (Figure 6B-6D) [49]. Taken as a whole, these results suggest that Cx37Hcs, Cx40Hcs, and Cx43Hcs mediate the sub-membranal Ca^2+ ^elevation responsible for eNOS activation, but not the bulk increase in [Ca^2+^]_i _caused by mechanical damage in surviving ECs. As the pharmacological profile of the decay phase is consistent with a CxHc pathway [2,26,27], it is conceivable that one or more additional Cx isoforms are expressed in rat aortic endothelium.

#### NO synthesis is abolished by caveolar disruption with MβCD

**Figure 7:**
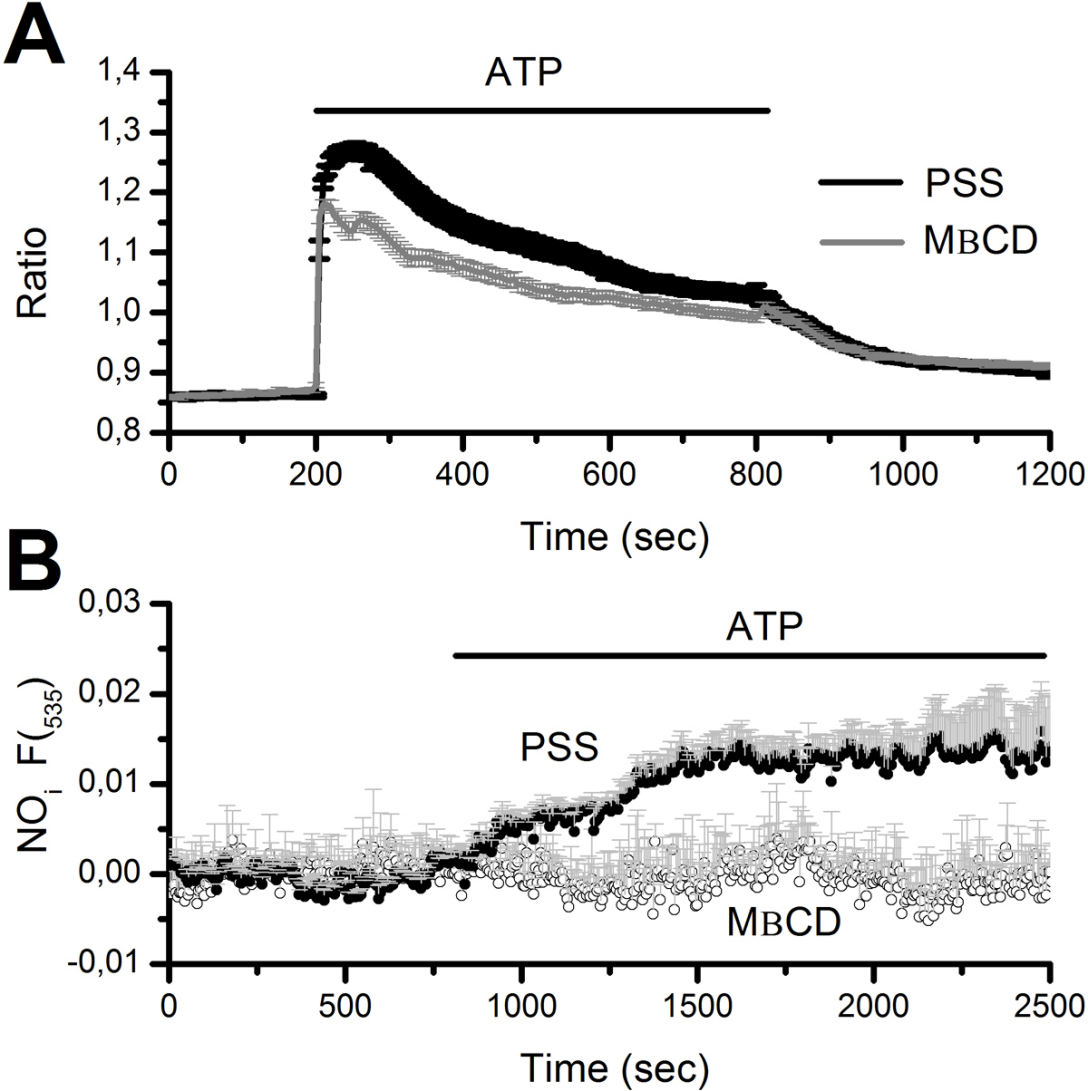
**Caveolar impairment impairs both the Ca^2+ ^response to ATP and the accompanying NO production in rat aortic endothelium**. A, pre-treatment with 10 mM MβCD to disrupt caveolae significantly hinders both the magnitude and the plateau of the Ca^2+ ^response to ATP (300 μM). Both phases depend on store-dependent Ca^2+ ^inflow when ATP is administered at this dose. The traces are the mean±SE of 220 (PSS, black trace) and 186 cells (MβCD, red trace), respectively, from no less than three different animals. B, MβCD (10 mM) suppresses ATP-induced NO synthesis. The traces are the mean±SE, respectively, of 150 (PSS, closed circles) and 4 (MβCD, open circles) cells from no less than three different animals.

**Figure 8:**
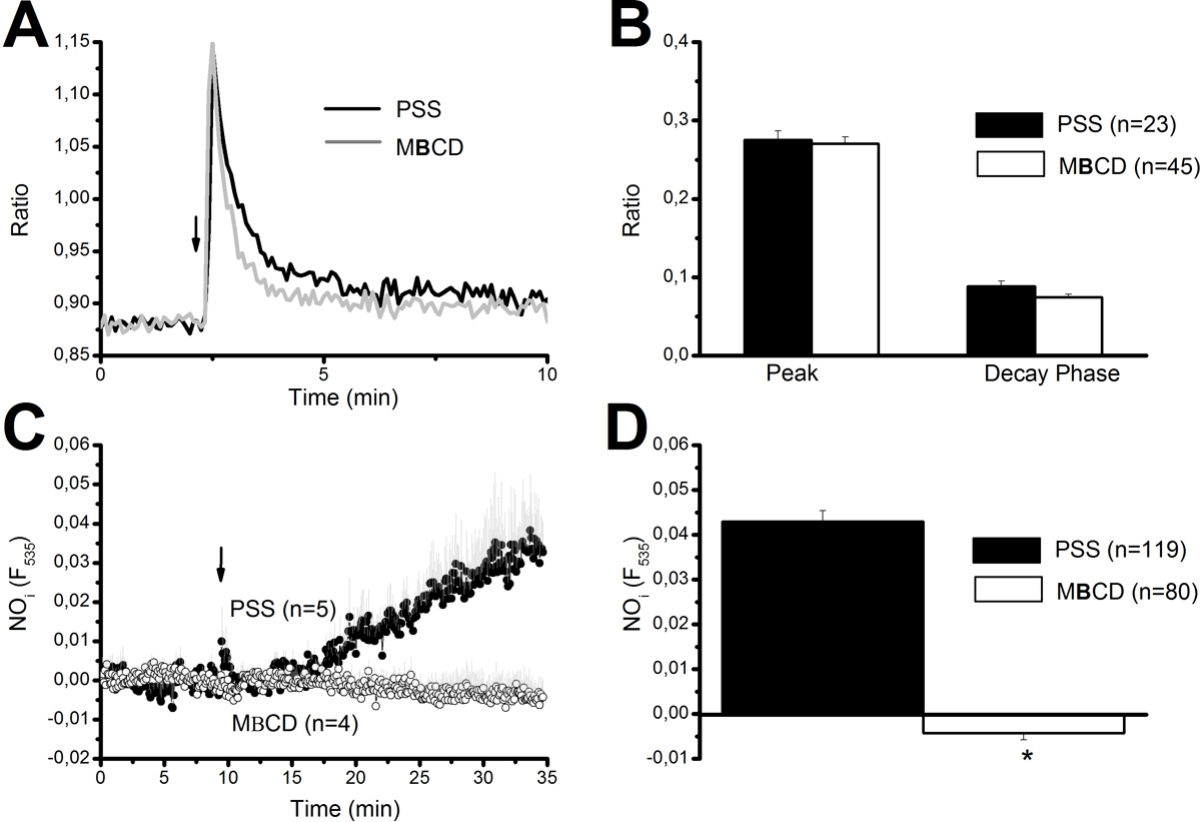
**Effect of caveolar impairment on injury-induced signalling in the luminal face of rat aorta**. Pre-treatment with 10 mM MβCD to disrupt caveolae does not significantly affect the Ca^2+ ^response to endothelial damage (A), but fully abolishes the accompanying NOP (C). B, MβCD effect on both the peak and the plateau of the Ca^2+ ^elevation. The traces are the mean±SE, respectively, of 5 (PSS, closed circles) and 4 (MβCD, open circles) cells from two aortic rings harvested from the same animal on the same day. D, MβCD effect on NOP amplitude measured at 20 min after the injury. For both B and D, the number of ECs analyzed in each condition is indicated above the bars.

NO production by a highly localized Ca^2+ ^signal sneaking beneath the plasma membrane requires the spatial proximity between the Ca^2+ ^source and eNOS [57,58]. Caveolae represent a signalling platform responsible for translating Ca^2+ ^entry into specific signal transduction pathways [24,57]. MβCD is a cholesterol-binding agent which has been widely employed to disrupt caveolar integrity in a variety of cell types, including ECs [24]. We first probed MβCD (10 mM) efficacy in impairing caveolae-dependent signalling by testing its effect on SOCE. As shown elsewhere [27,35], SOCE sustains the Ca^2+ ^response to high concentrations of ATP (300 μM) in the endothelium of excised rat aorta. As shown in Figure 7A, MβCD significantly reduced the amplitudes of both the initial Ca^2+ ^peak and the subsequent plateau, which are both supported by SOCE [27]. Consistently, MβCD inhibited ATP-dependent NOP in 85 out of 85 cells, whereas 93 out of 93 untreated cells liberated NO when exposed to ATP (Figure 7B). Then, to assess the role of caveolae in coupling CxHcs-mediated Ca^2+ ^inflow with eNOS activation, rat aortic rings were pre-incubated (30 min) in the presence of MβCD (10 mM). Exposition to MβCD did not significantly affect the Ca^2+ ^response to injury (Figure 8A and Figure 8B), but prevented the downstream NO synthesis (Figure 8C and Figure 8D). Collectively, these data indicate that the proximity between CxHcs and eNOS is maintained by the caveolar signalling platform within the plasma membrane. This feature is consistent with the highly localized nature of the Ca^2+ ^signal driving NO synthesis.

### Discussion

Endothelial-derived NO drives a number of processes which protects the vessel wall by pro-atherosclerotic events and maintain vascular homeostasis. NO exerts both an anti-aggregating and anti-inflammatory action, promotes endothelial survival, proliferation and migration, and prevents the phenotypic remodeling of the underlying layer of SMCs [2,19,59]. The present investigation provides the first evidence that endothelial ablation similar to that induced *in vivo *by stent deployment causes a local increase in NO levels by activating the ECs immediately adjacent to the lesion site. We further elucidated the molecular machinery responsible for eNOS recruitment and showed that injury-induced NOP is triggered by Ca^2+ ^entry though Cx37Hcs, Cx40Hcs, and Cx43Hcs. The coupling between Ca^2+ ^inflow and eNOS activation might be provided by cholesterol enriched membrane microdomains, such as caveolae. These data shed novel light on the endothelial reaction to mechanical scraping and hint at the Ca^2+ ^machinery as a novel target to restore endothelial integrity after reconstructive surgery and to treat vascular diseases [2,12].

#### NO synthesis in the injured endothelium of rat aorta is supported by CxHcs-mediated Ca^2+ ^entry

The procedure that we adopted for mechanical scraping mimics the partial denudation of the endothelial monolayer that has been described upon stent deployment in a number of pre-clinical models [41-43]. The extent of the lesion we inferred is smaller as compared to the degree of endothelial ablation observed after coronary stenting in humans in patients [60], albeit it is in the same range as that described in previous studies addressing the role of Ca^2+ ^signals in the regeneration of wounded monolayers [2,12]. However, a focal region devoid of the EC covering is rapidly repaired by neighbouring ECs, which spread into the lesioned area and undergo mitosis [61]. Accordingly, the daily rate of EC replication is increased by 50% at these sites in the rat aorta [62]. Therefore, the approach we utilized to perform the endothelial injury is useful to: 1) get insights into the acute response to the arterial damage in terms of Ca^2+ ^response and accompanying NO synthesis and 2) elucidate the molecular machinery driving the subsequent process of endothelial regeneration Three pieces of evidences suggest that injury-induced NO synthesis is mainly driven by Ca^2+ ^influx. First, there is no detectable NOP when the endothelium is scraped in absence of extracellular Ca^2+^. Second, injury-elicited NOP is not significantly affected by depletion of the InsP_3_-sensitive Ca^2+ ^stores with thapsigargin, although we cannot rule out the possibility that InsP_3_-sensitive NOP falls below the resolution of our fluorimetric system. Third, blockade of Ca^2+ ^entry by classic gap junction blockers, but not by BTP-2, dampen NO synthesis. These observations are consistent with the Fura-2 data and strongly suggest that CxHcs, rather than SOCE, provide the Ca^2+ ^source driving NO liberation in damaged endothelium. Rat aortic endothelium has been found to express three main Cx isoforms, namely Cx37, Cx40, and Cx43 [32,49]. In order to elucidate which of them underpins NO synthesis, we exploited the reported selectivity of Cx-mimetic peptides [33,45]. Both ^37,43^Gap27 and ^40^Gap27 significantly attenuated NOP triggered by Ca^2+ ^entry at the lesion site. The following lines of evidence confirm the specificity of ^37,43^Gap27 and ^40^Gap27: 1) pre-treatment with scrambled peptides did not detectably alter injury-induced NO synthesis (present study); and 2) ^37,43^Gap27 and ^40^Gap27 do not alter endogenous expression of Cx40 and Cx43 in the smooth muscle cell line A7r5, as well as connexin trafficking and *de novo *formation of gap plaques in A7r5 cells expressing Cx43-GFP [49]. These data strongly suggest that injury-elicited NOP at the wound edge of the aortic surface is sustained by Ca^2+ ^influx through Cx37Hcs, Cx40Hcs, and Cx43Hcs. These results are further supported by previous studies, which showed that Cx43-containing gap junctions mediate the spreading of pro-inflammatory Ca^2+ ^waves in lung capillaries [36] and sustain ATP-promoted Ca^2+ ^bursts in uterine artery ECs [63]. Consistent with the role served by CxHc as Ca^2+ ^entry pathways in vascular endothelium, Cx37Hc and Cx43Hc were recently shown to maintain bradykinin-induced intracellular Ca^2+ ^oscillations in rat brain endothelial cells [29], while Cx32Hc- and Cx43Hc-mediated Ca^2+ ^inflow supports the spiking response to bradykinin in Madin-Darby canine kidney cells [30]. Similarly, Cx43Hcs has already been shown to mediate Ca^2+ ^influx and activate Ca^2+^-dependent downstream targets in a variety of cell types other than endothelial cells [31,64].

#### Caveolar integrity is required to support injury-dependent NO synthesis in rat aortic endothelium

The subcellular analysis of the Ca^2+ ^signal driving eNOS activation has shown that Ca^2+ ^entry results in a highly localized Ca^2+ ^microdomain which is restricted to the inner leaflet of the plasma membrane [57,58]. Therefore, CxHcs and eNOS are likely to tightly interact in order for Ca^2+ ^inflow to stimulate NOP in injured endothelium. This hypothesis is supported by a number of recent studies. First, eNOS physically interacts with both Cx37 and Cx40 in *in situ *ECs of mouse aorta [65]. Second, Cx43 colocalizes with eNOS in native ECs covering the lumen of mice thoracodorsal arteries [66]. Third, Cx37 coimmunoprecipitates with eNOS in a number of human primary ECs [67]. The physical coupling between CxHcs and eNOS might be provided by cholesterol enriched membrane domains, such as caveolae. Indeed, MβCD abrogated injury-triggered NOP without affecting the underpinning Ca^2+ ^signal. This feature suggests that disruption of caveolae does not modify CxHs gating, but physically displaces eNOS from the local source of Ca^2+ ^responsible for its engagement. Consistently, caveolin-1 regulates the correct trafficking and localization to the plasma membrane of several Cx isoforms, including Cx 37, 40, and 43 [66,68,69]. Moreover, the expression of Cx37, Cx40 and Cx43 is decreased in caveolin-1 knock out arteries as compared to wild-type mice [70]. On the other hand, eNOS is targeted to caveolae by cotranslational N-myristoylation and posttranslational palmitoylation and interacts with the scaffolding domain of caveolin-1 [23]. It this, therefore, likely that caveolae maintain CxHcs in close proximity to eNOS and, perhaps, to other relevant signalling proteins, as recently suggested in [70]. Interestingly, while ineffective in regulating the Ca^2+^-mobilizing properties of CxHcs, caveolar integrity is key to SOCE recruitment in vascular ECs [57]. In line with these evidences, cholesterol depletion with MβCD prevents store-operated Ca^2+ ^inflow and NOP triggered by ATP in native rat aortic endothelium.

#### The putative role of NO in lesioned endothelium

The increase in NO levels at the wound edge represents an immediate, localized self-protecting response of the vessel wall to EC loss. Indeed, the NO signal is confined within the area experiencing the elevation in [Ca^2+^]_i _induced by the injury and does not spread farther away (see Figure 2C). EC detachment due to physiological turnover or pathological events causes the exposition of patches of sub-endothelial matrix which may activate platelets, a process known to promote an inflammatory reaction which favours the onset of atherosclerosis [71]. However, increased local NO synthesis inhibits both platelet aggregation and VSMC proliferation and migration, thus adversing both neointima formation and inflammation [1,19]. NO might play a key role also in subsequent healing [19,59]. Accordingly, NO inhibits apoptosis and stimulates proliferation and migration, which are an essential pre-requisite for ECs to spread and cover the de-endothelized area [19,59]. In injured rat aortic rings, both eNOS protein and enzyme activity have been shown to augment at the proliferating wound edge, but not in ECs distant from the lesion [25]. Moreover, aortic segments obtained from eNOS knock-out (KO) mice and cultured in Matrigel displayed a dramatic reduction in EC sprouting and proliferation as compared to wild-type animals [72]. Consistent with these *in vitro *observations, intravenous infusion of a NO donor accelerated *in vivo *regeneration of the endothelium lining the lumen of injured rat carotid artery [73]. In addition, neo-vascularization of ischemic hindlimbs, which depends on EC proliferation and migration, is significantly reduced in eNOS KO mice [74] and enhanced in eNOS overexpressing mice [75]. Finally, NO liberated by cells at the wound edge might recruit circulating endothelial progenitor cells and speed up the healing process [76].

The changes in DAF-FM fluorescence measured from the endothelial layer could also reflect some NOP occurring within the underlying SMCs, which generate a Ca^2+ ^wave in response to mechanical injury [48]. This Ca^2+ ^signal might, in turn, activate SMC eNOS and contribute to the increase in DAF-FM fluorescence we recorded from ECs at the wound edge [77]. However, injury-induced Ca^2+ ^waves in SMCs entirely depend on intracellular Ca^2+ ^release, rather than Ca^2+ ^influx [48]. As a consequence, the fact that all the protocols impairing injury-elicited Ca^2+ ^inflow into ECs nearby the lesion site (i.e. removal of external Ca^2+ ^and exposition to gap junction blockers) affected the accompanying increase in DAF-FM fluorescence strongly hint at the endothelial origin of the NO signal. Nevertheless, the possibility that NO synthesis also takes place within the tunica media of damaged rat aortic rings cannot be ruled out.

#### Evidence for the expression of additional Cx isoforms in the endothelium of rat aorta

The results presented in the current investigation support a role for Cx37Hcs, Cx40Hcs, and Cx43Hcs in driving the Ca^2+^-dependent eNOS activation at the wound edge of rat aortic endothelium. However, the decay phase of the Ca^2+ ^response to injury is not significantly affected by 1 hour pre-treatment with Cx-mimetic peptides. This feature concurs with the highly localized, sub-membranal nature of the Ca^2+ ^signal engaging eNOS in vascular ECs [57,58], which are detected by genetically-encoded Ca^2+ ^fluorophores selectively targeting the caveolae, but not the bulk cytoplasm [58]. This finding raises us to wonder about the molecular nature of the membrane pathway(s) responsible for the massive Ca^2+ ^influx observed during the decay phase. As widely discussed elsewhere [2,12], the pharmacological profile of such a route is typical of a CxHc. The lack of effect of Cx-mimetic peptides on the bulk increase in [Ca^2+^]_i _that features the decay phase of the Ca^2+ ^response to injury, therefore, suggests that Cx isoforms other than Cx 37, 40, and 43 are expressed in the native endothelium of rat aorta. This hypothesis is strongly supported by the recent discovery of Cx32 mRNA and protein in both cultured and blood vessel ECs [50]. Cx32Hcs, in particular, has recently been shown to mediate Ca^2+ ^influx when transfected in HeLa cells [78] and in Madin-Darby canine kidney cells [30]. Additional Cx isoforms that have been implicated in Ca^2+ ^entry across the plasmalemma are Cx26 [79] and Cx45 [80]. That Cx isoforms other than Cx37, Cx40, and Cx43 are expressed in the vessel wall has also been proposed by Tang and Vanhoutte [52] and by Sorensen et al. [81]. Both groups reached the same conclusion upon the finding that connexin mimetic peptides failed to reproduce the effects of classic gap junction blockers in the vasculature. Recent evidence hinted at pannexins (Pxs), which are hexameric channels structurally unrelated to Cxs, as the molecular substrates for the hemichannels (the so-called pannexons) present in the non-junctional area of cell membrane [82]. However, to the best of our knowledge, Px expression has hitherto been reported only in microvascular beds rather than in macrovessels, such as aorta [82]. Moreover, as recently pointed out [2,12], Px hemichannels are unaffected by La^3+ ^and heptanol, which block CxHcs and impair injury-induced Ca^2+ ^entry in the intact endothelium of rat aorta [26,27]. Accordingly, our preliminary data indicate that probenecid, a widely employed Px blocker, does not significantly affect the Ca^2+ ^response to mechanical injury in rat aortic endothelium. It is worth of noting that Cx-mimetic peptides, either separately or in combination, caused an increase in the InsP_3_-dependent initial Ca^2+ ^peak. A recent study reported that CxHcs may mediate InsP_3 _efflux into the extracellular space [83]. It is, therefore, conceivable that the prolonged pre-treatment with Cx-mimetic peptides prevented InsP_3 _produced at the wound edge from moving out of the cytosol, thus increasing the extent of Ca^2+ ^release in the first raw of cells.

## Conclusion

In conclusion, this study provides the first clear-cut evidence that arterial damage activates surviving ECs to produce NO by inducing Ca^2+ ^entry via Cx37Hcs, Cx40Hcs, and Cx43Hcs. Our data indicate that the membranal compartmentalization of CxHcs and eNOS depends on is enabled by caveolae. Experiments are under way in our laboratory to assess the role played by CxHcs-induced NO synthesis in the regeneration of rat aortic endothelium. These results outline the importance of designing novel prosthetic devices able to release NO after vascular interventions, such as arterial bypass grafting and intravascular stent deployment [84]. Indeed, the currently employed drug-eluting stents may result in late arterial occlusion and, eventually, in patient death due to their reported capability to inhibit endothelial regeneration [11]. Alternatively, the signalling cascade leading to NO production that we have described in the present work might be the alternative target of novel drug-eluting stents. The cardiological practice has unveiled a variety of compounds, such as ZP123 or rotigaptide and (2S,4R)-1-(2-aminoacetyl)-4-benzamidopyrrolidine-2-carboxylic acid hydrochloride (GAP-134), which increase CxHc conductance and, therefore, might be exploited to increase Ca^2+ ^influx at the lesion site [2,12]. These substances have successfully been probed as orally-active and anti-arrhythmic drugs in Phase I and II clinical trials conducted on patients suffering either from atrial fibrillation or from unstable angina or myocardial infarction [85,86]. In perspective, these molecules might be locally released by drug-eluting stents to improve CxHc-mediated signalling and boost NO synthesis after surgical interventions on diseased vessels [2,12].

## Competing interests

The authors declare that they have no competing interests.

## Authors' contributions

RBR: conceived the study, carried out the experiments, analyzed and interpreted the data, drafted the manuscript. JEAC: conceived the study, carried out the experiments, analyzed and interpreted the data. AR: conceived the study, carried out the experiments, analyzed and interpreted the data. ADC: critically revised the manuscript. MPC: critically revised the manuscript. SM: critically revised the manuscript. GG: conceived the study and critically revised the manuscript. FM: conceived the study, analyzed and interpreted the data and drafted the manuscript. FT: conceived the study, analyzed and interpreted the data, critically revised the drafted manuscript. All authors read and approved the final manuscript.

## Authors' information

RBR: Professor of Physiology at Benemérita Universidad Autónoma de Puebla. JEAC: Researcher at University of Pavia. AR: Postdoctoral Fellow at University of Calgary. ADC: Assistant Professor of Cardiac Surgery at Second University of Naples. MPC: Assistant Professor of Anatomy at University of Naples "Federico II". SM: Full Professor of Anatomy at University of Naples "Federico II". GG: Assistant Professor of Anatomy at University of Molise. FM: Assistant Professor of Physiology at University of Pavia. FT: Associate Professor of Physiology at University of Pavia.

## Declarations

Publication charges for this article were covered by research funds of the project Bando Faro 2012 - Finanziamenti per l'Avvio di Ricerche Originali, cofounded by the Compagnia di San Paolo and by the Polo per le Scienze e le Tecnologie per la Vita of the University Federico II in Naples.

This article has been published as part of *BMC Surgery *Volume 13 Supplement 2, 2013: Proceedings from the 26th National Congress of the Italian Society of Geriatric Surgery. The full contents of the supplement are available online at http://www.biomedcentral.com/bmcsurg/supplements/13/S2
